# Two Pandemics, One Challenge—Leveraging Molecular Test Capacity of Tuberculosis Laboratories for Rapid COVID-19 Case-Finding

**DOI:** 10.3201/eid2611.202602

**Published:** 2020-11

**Authors:** Susanne Homolka, Laura Paulowski, Sönke Andres, Doris Hillemann, Ruwen Jou, Gunar Günther, Mareli Claassens, Martin Kuhns, Stefan Niemann, Florian P. Maurer

**Affiliations:** National and Supranational Reference Centre for Mycobacteria, Research Centre Borstel, Borstel, Germany (S. Homolka, L. Paulowski, S. Andres, D. Hillemann, M. Kuhns, S. Niemann, F.P. Maurer);; Molecular and Experimental Mycobacteriology Unit, Research Centre Borstel, Borstel (S. Homolka, S. Niemann);; Diagnostic Mycobacteriology Unit, Research Centre Borstel, Borstel (L. Paulowski, S. Andres, D. Hillemann, M. Kuhns, F.P. Maurer);; National Reference Laboratory of Mycobacteriology, Centres for Disease Control, Ministry of Health and Welfare, Taipei, Taiwan (R. Jou);; University of Namibia School of Medicine, Windhoek, Namibia (G. Günther, M. Claassens);; Inselspital, Bern University Hospital, University of Bern, Bern, Switzerland (G. Günther);; German Center for Infection Research, Research Centre Borstel, Borstel (S. Niemann);; University Medical Centre Hamburg-Eppendorf, Hamburg, Germany (F.P. Maurer)

**Keywords:** COVID-19, coronavirus disease, SARS-CoV-2, severe acute respiratory syndrome coronavirus 2, viruses, respiratory infections, zoonoses, tuberculosis and other mycobacteria, bacterial infections, laboratory, PCR, quality control, bacteria

## Abstract

In many settings, the ongoing coronavirus disease (COVID-19) pandemic coincides with other major public health threats, in particular tuberculosis. Using tuberculosis (TB) molecular diagnostic infrastructure, which has substantially expanded worldwide in recent years, for COVID-19 case-finding might be warranted. We analyze the potential of using TB diagnostic and research infrastructures for severe acute respiratory syndrome coronavirus 2 (SARS-CoV-2) testing. We focused on quality control by adapting the 12 Quality System Essentials framework to the COVID-19 and TB context. We conclude that diagnostic infrastructures for TB can in principle be leveraged to scale-up SARS-CoV-2 testing, in particular in resource-poor settings. TB research infrastructures also can support sequencing of SARS-CoV-2 to study virus evolution and diversity globally. However, fundamental principles of quality management must be followed for both TB and SARS-CoV-2 testing to ensure valid results and to minimize biosafety hazards, and the continuity of TB diagnostic services must be guaranteed at all times.

The ongoing coronavirus disease (COVID-19) pandemic presents a massive challenge for healthcare systems globally (*1*,*2*). Rapid case-finding and patient isolation are crucial to limit transmission and avoid exceeding capacity limits of critical healthcare infrastructures. Therefore, the World Health Organization (WHO) (*3*) strongly advocates a large and rapid increase of global testing capacities to detect severe acute respiratory syndrome coronavirus 2 (SARS-CoV-2) RNA (*2*,*4*). This task is enormous, in particular in resource-poor settings without widespread availability of microbiological laboratories and, even more so, specialized virologic laboratories. For example, although other outbreaks such as the 2014–2016 Ebola epidemic in West Africa triggered substantial investments into surveillance and preparedness, many hospitals, clinics, and laboratories in sub-Saharan Africa were already operating at maximum capacity before the COVID-19 pandemic (*5*). Consequently, the WHO Joint External Evaluation reports suggest that the ability to respond to an international health hazard, such as the importation of an infectious disease like COVID-19, requires almost universal laboratory improvement across sub-Saharan Africa (*6*).

With one quarter of the world’s population infected with bacteria belonging to the *Mycobacterium tuberculosis* complex (MTBC), tuberculosis (TB) still represents a major global health threat (*7*). Substantial efforts have been made to scale-up highly sensitive and specific molecular diagnostic systems in high- and low-resource settings, which greatly improved TB care globally (*8*). Strategies included programmatic implementation of (near) point-of-care, easy-to-handle testing systems, such as the cartridge-based GeneXpert (Cepheid, https://www.cepheid.com) platform (*8*,*9*). In addition, high-throughput PCR instruments are in use mostly at large central laboratories, in particular those also offering HIV and hepatitis viral load testing (*10*). Because TB diagnosistic infrastructures offer high spatial coverage, preexisting supply chains and clinical networks, staff trained to work with airborne pathogens, and the availability of analytical and biosafety equipment, leveraging the potential of these systems for SARS-CoV-2 testing is tempting. In fact, guidance has recently been issued by WHO (*11*) and the Stop TB Partnership (*7*). However, although rapid action is needed, we believe that quality must not be sacrificed for speed. We examined what efforts are needed to allocate molecular testing capacity to SARS-CoV-2 case-finding and research in laboratories usually dealing with TB and how the essentials of quality control apply in this context.

## Establishing SARS-CoV-2 Testing Capacity for Routine Patient Care

We studied the availability of SARS-CoV-2 assays on analytical platforms commonly used in TB laboratories of different service levels, that is, at the point of care, at peripheral testing sites, at intermediate laboratories, and at central laboratories (Appendix Table 1) (*12*). It is evident that these approaches differ considerably with respect to setting, sample throughput, and hands-on time. For designing a SARS-CoV-2 testing strategy for a TB laboratory, we suggest considering 5 aspects: availability of hardware and consumables, expected throughput, distance between sampling sites and the laboratory, available personnel and their qualification levels, and pricing. For example, clearly a rapid testing response would ideally rely on existing instruments. In this regard, using GeneXpert instruments, which are available at many TB laboratories, is an obvious consideration. In addition, the Xpert Xpress SARS-CoV-2 testing cartridge, which has received US Food and Drug Administration (FDA) emergency use approval, is available through the Global Drug Facility of the Stop TB Partnership, albeit with a lead time of several months and at a price of $19.80 USD, which might still be too high to allow high-throughput testing (*13*,*14*). Also, in many district laboratories, single 4-slot GeneXpert instruments are used for TB diagnostics, which will easily be overwhelmed by a community screening program for SARS-CoV-2, putting at risk both COVID-19 and TB response. Moreover, although prior experience with GeneXpert is beneficial, additional training on interpretation of the Xpert SARS-CoV-2 assay results will be required. For example, unlike Xpert MTB/RIF, the Xpert SARS-CoV-2 assay might yield a presumptive positive result calling for a reflex testing algorithm made available for such cases. Furthermore, in some low-resource settings, PCR-based tests are still only available at central laboratories far away from primary or secondary healthcare facilities. These potential complications would increase turnaround time even with a relatively quick and easy test. Laboratories intending to offer SARS-CoV-2 testing must therefore thoroughly evaluate whether the available instruments will meet current and expected demands. This task is challenging because sample numbers might quickly increase, for example, because of the dynamics of the epidemic or through changes in testing policies, or decrease, for example, because of additional laboratory capacity becoming available elsewhere.

Similar considerations apply to staffing resources. Although in-house PCRs are comparably cheap and flexible because PCR chemistry of different manufacturers can be used, they are technically more demanding to perform than cartridge-based tests. Rigorous process control and higher operator skill levels are required to minimize cross-contamination, sample mixups, and PCR failures. In contrast, cartridge-based systems offer ease of use and rapid results at the cost of being dependent on a single manufacturer for reagent resupplies and instrument maintenance. Because countries have been bidding against each other for limited test reagents, low-resource countries with limited local funding might have concerns about their ability to procure enough tests. WHO, together with the United Nations and other international organizations, have recently set up a Global Supply Chain Task Force to secure SARS-CoV-2 tests produced by several manufacturers at negotiated prices for low- and middle-income countries (*3*).

Consideration of changing testing demands for TB during the ongoing SARS-CoV-2 pandemic also is important. For instance, at a national reference laboratory level, we experienced a decrease in samples sent for culture-based TB testing during the 12-week period of mid-March through mid-June, whereas requests for molecular TB testing increased. This pattern is likely because peripheral laboratories focused their own capacities on SARS-CoV-2 PCRs. Laboratory managers tasked to allocate workforce to SARS-CoV-2 testing need to consider potentially changing TB testing demands to guarantee the uninterrupted availability of TB diagnostic services at all times.

## Biosafety and the 12 Quality System Essentials

Achieving, maintaining, and improving accuracy, timeliness and reliability of test results are key deliverables of diagnostic laboratories. As is the case for TB, late or false-negative SARS-CoV-2 test results will lead to delays in or even preclude correct diagnosis, jeopardizing timely isolation and prevention of transmission. In turn, false-positive tests will waste public health resources, will lead to incorrect epidemiologic data, and might even lead to patient stigmatization. Quality control is a cornerstone of safe, consistent, reliable diagnostics, and many studies and frameworks outline the structure of quality-management systems suitable for diagnostic laboratories (*15**–**18*).

We used the laboratory quality-management system guidance issued jointly by WHO, the US Centers for Disease Control and Prevention, and the Clinical and Laboratory Standards Institute to deduce critical interventions and management tasks required to expand the diagnostic workflow of TB laboratories to SARS-CoV-2 testing in a quality-controlled manner (*18*). Based on International Organization for Standardization document 15189 and Clinical and Laboratory Standards Institute document GP26-A3, the 12 Quality System Essentials approach is centered on 12 interlinked topics: organization, personnel, equipment, purchasing and inventory, process control, information management, documents and records, occurrence management, assessment, process improvement, stakeholder service, and facilities and safety. All 12 topics have practical implications relevant to the context of successfully implementing SARS-CoV-2 testing in TB laboratories (Appendix Table 2). When considering the 12 Quality System Essentials, we found that making the necessary changes to the analytical workflow is just one piece in the puzzle. In fact, several additional steps are needed, ranging from staff training (e.g., on sample collection for COVID-19 testing, which differs from coaching patients to produce sputum, and the definition and review of meaningful quality indicators) to participation in SARS-CoV-2 proficiency testing and anticipation of strategies for management of nonconformities. Furthermore, biosafety procedures will need to be carefully scrutinized, and staff instructions will need to be adapted in a concise, practical, and easy-to-understand manner. Procedures such as performing virus propagation, virus isolation, or neutralization assays should be performed only by competent personnel under Biosafety Level 3 conditions, ruling out such work at peripheral laboratories and in many resource-poor settings. WHO has summarized its biosafety recommendations for working with SARS-CoV-2 (*19*).

## Using TB Infrastructure for Research on SARS-CoV-2

Although research requiring propagative work with SARS-CoV-2 will likely be beyond the scope of most dedicated TB laboratories, even when equipped with fully operational Biosafety Level 3 facilities, the TB community is strongly influenced by progress in next-generation sequencing (NGS), a technology that is also in heavy demand for research on SARS-CoV-2. Prime examples for the application of NGS in the TB field are the prediction of drug resistance from genome sequencing of clinical isolates (which can potentially also be performed directly from clinical samples), evolutionary studies looking into the adaptation of MTBC strains in response to antibiotic treatment, and the use of genome sequencing to trace local, regional, and national transmission or for cross-border molecular surveillance (*8*,*20*–*23*). Over the past few years, the potential of NGS technologies to replace time-consuming and complex-to-perform phenotypic techniques for resistance testing of MTBC isolates became more evident (*21*,*24*). Accordingly, WHO has released a technical guide on the use of NGS for the detection of resistance-associated mutations in MTBC strains (*25*), and some countries, such as the United Kingdom, have already shifted their TB diagnostic and surveillance approach to NGS, including substantial investments in hardware and bioinformatics infrastructure (*26*). In addition, NGS facilities are increasingly established in settings with high TB prevalence, including implementation of laboratory workflows with data analysis pipelines and quality-control procedures, in line with the 12 Quality System Essentials (*18*). In parallel to workflow and infrastructure set up, intensive training of technical and academic personnel is ongoing, for example, through a network dedicated to the application of sequencing technologies for the fight against resistant TB in high-incidence settings (SeqMDR TB_NET), which supports the implementation of NGS technologies in Kyrgyzstan, Moldova, Namibia, Mozambique, and Eswatini (https://ghpp.de/en/projects/seqmdrtb-net). These sequencing capacities, which are embedded in local and international clinical and epidemiologic research networks, are in principle suited to address urgent research questions related to the COVID-19 epidemic, such as establishing key epidemiologic, clinical, and virologic characteristics of the pathogen and, in particular, defining its ability to spread in humans. Several sequencing protocols, such the ARTICnetwork nCoV-2019 protocol (https://artic.network/ncov-2019), have been developed for NGS of SARS-CoV-2 (*27*). Virus sequencing has been used early in the epidemic to understand the origin, spread, and evolution of SARS-CoV-2 in different regions of the world as well as for outbreak investigations (*28*–*31*). SARS-CoV-2 sequences are also collected by online tools that enable a prospective monitoring of the virus spread and evolution on the global level. For example, 4,397 genomes sampled during December 2019–August 2020 are archived in the GISAID online hCoV-19 database (https://www.gisaid.org/epiflu-applications/next-hcov-19-app). Virus sequencing will be crucial in the next phase of the COVID-19 pandemic for population-based surveillance and control of viral transmission (e.g., by allowing a precise understanding of the regional spread of the virus in relation to time, place, human migration, and other determinants) (*32*).

Another important aspect is the influence of co-infection with TB (and also with HIV) on the epidemiologic, clinical, virologic and immunological trajectory of COVID-19, and vice versa, in high-incidence settings as we observe the collision of 3 global pandemics with unpredicted outcomes. Moreover, with a renewed global focus on active case-finding in TB programs, resources dedicated for COVID-19 community-based research, such as household contact tracing or seroprevalence surveys, could easily be linked to programs to test for TB as well, providing a gateway for training, capacity building, and future TB research. However, as is the case for the capacity of TB diagnostic services, careful planning and close collaboration between the TB, HIV, and COVID-19 research communities will be crucial not to overburden these infrastructures, especially in resource-poor settings. In addition, strong political will and support for research communities are essential, especially in low- and middle-income settings, to advocate for and allocate resources needed to investigate these coinciding pandemics.

## Conclusion

TB laboratories can be an important resource to increase the global capacity for SARS-CoV-2 diagnostic testing and research. However, expanding their scope to the detection of a viral pathogen warrants careful planning. Challenges will be different for peripheral, intermediate, and central-level laboratories and can relate to any of the 12 Quality System Essentials we have outlined. Despite the availability of SARS-CoV-2 assays on all major molecular TB testing systems, careful capacity planning is crucial to match the local demand, operator skills, and funding available. Mitigating the risk for supply chain interruptions is another key management task, and establishing >1 SARS-CoV-2 test is advisable to guarantee service continuity. The diagnostic industry is challenged to manufacture SARS-CoV-2 tests without deprioritizing production of reagents needed to test for TB, HIV, and malaria. In addition, concerns exist about TB case-finding and culture-based diagnostics being impaired by the ongoing SARS-CoV-2 pandemic, as has been shown in a rapid assessment by the Stop TB Partnership (*33*). Likewise, a recent modeling analysis showed a 70% drop in the probability of TB diagnosis per visit to a healthcare provider because of reduced laboratory capacity and availability of healthcare staff secondary to the COVID-19 pandemic in countries such as India, Kenya, and Ukraine (*34*). Consequently, although leveraging the globally available TB diagnostic and research infrastructures is a powerful strategy to increase SARS-CoV-2 testing capacity and to elucidate some of the open research questions that have arisen during the ongoing SARS-CoV-2 pandemic, care must be taken that TB services are not disrupted at any time during the COVID-19 response.

Appendix Additional information about leveraging molecular test capacity of tuberculosis laboratories for rapid COVID-19 case-finding.
