# Supplementary material for: Two Pandemics, One Challenge—Leveraging Molecular Test Capacity of Tuberculosis Laboratories for Rapid COVID-19 Case-Finding
Source: Emerg Infect Dis. 2020 Nov;26(11):2549–54. doi: 10.3201/eid2611.202602 (PMC7588527; doi:10.3201/eid2611.202602)
Supplement: Appendix — Additional information about leveraging molecular test capacity of tuberculosis laboratories for rapid COVID-19 case-finding. [file 20-2602-Techapp-s1.pdf]

Article DOI: https://doi.org/10.3201/eid2611.202602

Two Pandemics, One Challenge—Leveraging Molecular Test Capacity of Tuberculosis Laboratories for Rapid COVID-19 Case-Finding

Appendix

Appendix Table 1. Platforms commonly available at TB testing sites suitable for detection of SARS-CoV-2 RNA*

System	Manufacturer	Laboratory level (*1*)	SARS-CoV-2 assay available	Throughput (samples/time)	Recommended specimen type	Approval status of SARS-CoV-2 assay	Comment
GeneXpert	Cepheid	PL, IML, CL	Yes	1 to 80/45 min (depending on instrument)	NP swabs	FDA-EUA	Approximately 23,000 systems deployed worldwide†; SARS-CoV-2 cartridges available through the Stop TB Partnership Global Drug Facility
Truenat Beta CoV	Molbio Diagnostics	PL, IML, CL	Yes	4/1 h		CE-IVD	Confirmatory SARS-CoV-2 test recommended
BD MAX	Becton Dickinson	PL, IML, CL	Yes	24/3 h	NP and OP swabs	FDA-EUA, CE-IVD	
Manual or semiautomatic NA extraction combined with programmable realtime PCR thermocyclers	Various, e.g., Applied Biosystems 7500; Bio-Rad CFX ConnectTM; Roche LightCycler 480 II; Qiagen Rotor-Gene Q	IML, CL	Yes	up to 96/3 h‡	NP and OP swabs, sputum	Depending on reagents used§	Both commercial reagent kits and in-house methods available
Cobas 6800/8800	Roche	CL	Yes	384/8 h; 1056/8 h	NP swabs	FDA-EUA, CE-IVD, WHO-EUL	
m2000	Abbott	CL	Yes	470/24 h	NP and OP swabs	FDA-EUA, CE-IVD, WHO EUL	

*PL, peripheral laboratory; IML, intermediate laboratory; CL, central laboratory; NP, nasopharyngeal; OP, oropharyngeal; FDA, United States Food and Drug Administration; EUA, Emergency Use Authorization; WHO, World Health Organization; EUL, Emergency Use Listing; NA, nucleic acid; CE-IVD, *Conformité Européenne-in vitro* diagnostic.
†According to manufacturer (*2*).
‡Depending on RNA extraction protocol and thermocycler used.
§Various CE-marked kits available; some PCR assays are included in WHO emergency use listing for in vitro diagnostics detecting SARS-CoV-2 nucleic acid (*3*).

Appendix Table 2. Key determinants for achieving, maintaining and improving accuracy, timeliness and reliability of laboratory test results and their implication for SARS-CoV-2 testing in TB laboratories*

Determinant	Description	Implementation strategies for the SARS-CoV-2 / MTBC context
Organisation	• Existence of a formal QMS that supports consistent procedures<br>• The management team and quality unit play an integral role in a quality-driven culture, along with structures for monitoring ongoing quality	• Expand the scope of the laboratory quality management system to SARS-CoV-2 testing<br>• Define scope and assign clear responsibilities for both implementation of SARS-CoV-2 testing and maintenance of TB diagnostic service<br>• Set up a regular SARS-CoV-2 briefing<br>• Laboratory leadership needs to implement an internal communication strategy to assure adequate information of staff on SARS-CoV-2 pathobiology, biosafety in relation to MTBC, changes in laboratory organization, prioritisation of MTBC versus SARS-CoV-2 testing<br>• It is critical that TB services are not disrupted during the COVID19 response
Facilities and safety	• Laboratories need a comprehensive set of procedures and standards to ensure a safe, secure, and clean environment	• Define SARS-CoV-2 workspaces and usage times for shared equipment to minimize interference with TB diagnostics<br>• Limit SARS-CoV-2 laboratory access to authorized staff<br>• Place orders for additional PPE with >1 distributor to mitigate risk of shortages<br>• Check whether available disinfectants have proven activity against enveloped viruses<br>• Implement a staff screening mechanism for COVID-19 symptoms (some TB laboratories in high-burden settings routinely screen staff with a TB symptom questionnaire)
Equipment	• Every piece of equipment used in the laboratory must be maintained to assure correct operation	• Assess if additional equipment is needed for SARS-CoV-2 testing<br>• Develop contingency plans for equipment failures, if possible set-up >1 SARS-CoV-2 assay to mitigate risk of reagent shortages<br>• Check maintenance protocols for pipets, UV clean spots, safety cabinets, thermocyclers, and freezers
Purchasing and inventory	• Proper supply chain management is critical to ensure that raw inputs and other supplies are consistently available and of high quality<br>• Inventory activities should verify that materials and supplies are stored in a way that protects integrity	• Establish clear processes and responsibilities for selection, purchasing, order tracking and storage of SARS-CoV-2 supplies<br>• Documentation and daily review of order status and inventory for SARS-CoV-2 reagents, MTBC reagents and PPE<br>• Develop contingency plans and allocate ressources for supply chain disruptions for all critical consumables, e.g., quality-controlled in-house preparation of transport medium, running alternative assays, reducing testing frequency, diverting samples to other laboratories
Process control	• Process control encompasses QC processes for testing	• Perform in-house assay verification / validation for all newly introduced methods and reagents<br>• Ensure that extraction and amplification controls, positive and negative control samples as well as QC ranges are valid for each test run before release of patient results<br>• Set up lot control documentation for all SARS-CoV-2 test reagents<br>• Implement four-eyes principle for interpretation and release of SARS-CoV-2 test results<br>• Verify that the in-house testing algorithm is consistent with national and international standards and technical guidelines
Assessment	• Systematic examination of the quality management system to demonstrate that testing meets regulatory, accreditation and customer requirements	• Cross-check random samples in regional, national or international SARS-CoV-2 reference laboratories<br>• Set up internal and external SARS-CoV-2 audits

Determinant	Description	Implementation strategies for the SARS-CoV-2 / MTBC context
		• Participate in SARS-CoV-2 proficiency testing
Personnel	• Training, motivation, and engagement of staff members as key parts of quality-controlled diagnostics	• Assure competency of personnel involved in SARS-CoV-2 testing by defining a practical training schedule with documented assessment including, as needed, safe sample collection, handling, transport, disposal of swabs, nucleic acid preparation, instrument operation, handling of results, biosafety<br>• Develop contingency plans for staff shortages<br>• Participation of key personnel in COVID-19 webinars can support rapid knowledge transfer to a local group of experts<br>• Perform regular staff briefings on the local, national and international development of the COVID-19 situation to maintain motivation and engagement
Stakeholder service	• The laboratory needs to understand the stakeholders and their needs and use feedback for improvement	• Identify the needs of clinicians with regard to specimen transport, turnaround times for inpatients and outpatients, and reporting preferences<br>• Identify the needs of public health authorities with respect to case notification requirements
Occurrence management	• Correct handling of nonconformities / accidents	• Document and review all occurrences followed by feedback and discussion with technical staff<br>• Facilitate investigations to identify the root cause of any occurrence to prevent reoccurrence
Process improvement	• Process improvement establishes a program to ensure continuous quality improvement over time	• Define meaningful (measurable, achievable, interpretable, actionable, balanced, timed) quality indicators for SARS-CoV-2 testing, e.g., for turnaround time, competency of personnel, quality control, proficiency testing and customer satisfaction<br>• Provide regular feedback to personnel about test and QC results<br>• Foster team discussion of unclear results
Documents and records	• Documents need to be available at the point of work, maintained, accurate, and secure	• Establish an SOP for SARS-CoV-2 testing covering reagent and sample management (collection, transport, processing, storage, retention, disposal), testing procedure and information management (reporting, notification to health authorities, archiving)<br>• Set up a SARS-CoV-2 laboratory report sheet<br>• Implement supporting documentation, e.g., training checklists, briefing protocols, inventory spreadsheets<br>• Implement SARS-CoV-2 document control and storage
Information management	• Laboratory data needs to be managed in a way that ensures all information is accurate, secure, confidential, and accessible to individuals with the right privileges	• Expand the current TB laboratory information workflow to handle SARS-CoV-2 data<br>• Implement a process for timely notification of public health authorities

*QMS, quality management system; MTBC, *Mycobacterium tuberculosis* complex; PPE, personal protective equipment; SOP, standard operating procedure; UV, ultraviolet. Adapted from WHO guidelines (*4*).

References

1. World Health Organization. Laboratory services in TB control, part I: organization and management. Geneva: The Organization; 1998 [cited 2020 May 6]. https://apps.who.int/iris/bitstream/handle/10665/65942/WHO_TB_98.258_(part1).pdf

2. Cepheid. Cepheid | Xpert® Xpress SARS-CoV-2 has received FDA Emergency Use Authorization. 2020 [cited 2020 May 6]. https://www.cepheid.com/coronavirus

3. World Health Organization. WHO emergency use listing for in vitro diagnostics (IVDs) detecting SARS-CoV-2 nucleic acid. 2020 May 11 [cited 2020 May 14]. https://www.who.int/diagnostics_laboratory/200511_eul_sars_cov2_product_list.pdf

4. World Health Organization. Laboratory quality management system: handbook. Geneva: The Organization; 2011 [cited 2020 May 6]. https://www.who.int/ihr/publications/lqms
